# The Impact of Acute Loud Noise on the Behavior of Laboratory Birds

**DOI:** 10.3389/fvets.2020.607632

**Published:** 2021-01-06

**Authors:** Tayanne L. Corbani, Jessica E. Martin, Susan D. Healy

**Affiliations:** ^1^The Royal (Dick) School of Veterinary Studies, The College of Medicine and Veterinary Medicine, The University of Edinburgh, Edinburgh, United Kingdom; ^2^The Royal (Dick) School of Veterinary Studies and The Roslin Institute, The College of Medicine and Veterinary Medicine, The University of Edinburgh, Edinburgh, United Kingdom; ^3^School of Biology, Harold Mitchell Building, University of St. Andrews, St. Andrews, United Kingdom

**Keywords:** noise stress, avian husbandry, zebra finch (*Taeniopygia guttata*), bird, passerine, animal welfare

## Abstract

Husbandry procedures and facility settings, such as low-frequency fire alarms, can produce noises in a laboratory environment that cause stress to animals used in research. However, most of the data demonstrating harmful effects that have, consequently, led to adaptations to management, have largely come from laboratory rodents with little known of the impacts on avian behavior and physiology. Here we examined whether exposure to a routine laboratory noise, a low-frequency fire alarm test, induced behavioral changes in laboratory zebra finches (*Taeniopygia guttata*). Twenty-four breeding pairs of zebra finches were randomly selected and exposed to the low-frequency fire alarm (sounding for 10–20 s) or no noise (control) on separate test days. All birds were filmed before and after the alarm sounded and on a control day (without the alarm). The zebra finches decreased their general activity and increased stationary and social behaviors after exposure to the alarm. Brief exposure to a low-frequency alarm disrupted the birds' behavior for at least 15 min. The induction of this behavioral stress response suggests that low-frequency sound alarms in laboratory facilities have the potential to compromise the welfare of laboratory birds.

## Introduction

The ethics of using animals in research have long been an issue (1–3). More recently the reproducibility of animal research outcomes has also raised concerns (4–8) because the physical and social environment of the laboratory provide significant sources for a range of stimuli that can influence an animal's physiology and behavior, its welfare, and scientific outcomes (9). A number of these concerns focus on variable holding conditions across research facilities (10–12). For example, factors such as lighting, temperature and husbandry procedures can introduce variability as well as being potentially stressful (9, 10, 13, 14). Standardization of laboratory conditions such as using sets of defined acceptable ranges (e.g., for lighting, temperature and so on: (15) is one way in which researchers attempt to decrease the impacts of such confounding variables (16, 17).

But the acoustic environment and noise levels are among those aspects to which little attention has been paid. Although noise is considered a potentially stressful factor for both humans and animals (18–22), there is still a lack of awareness and appreciation of the degree to which laboratory animals might be affected by the environmental noise and how that might impact experimental outcomes (23).

Although the concern about noise in animal laboratory facilities was raised decades ago (24) and despite recent efforts to emphasize the scale of the problem (10, 25, 26), comprehensive changes are yet to be seen in laboratory and husbandry procedures: the soundscape of modern animal facilities has changed little over the past 10–20 years (27). Furthermore, descriptions of environmental noise conditions are not requested in the publication guidelines of at least nine major journals that publish research based on animal experimentation (28), while other conditions such as temperature, humidity and light cycle are obligatory for publication in at least one of these journals (28). As a result acoustic conditions are seldom reported in research communications, in contrast to other environmental parameters such as lighting, temperature, and humidity (25).

In the laboratory environment noises are produced from a range of sources including husbandry procedures and facility hardware (25, 27, 29–31). Elevated sound pressures exerted by such features can cause a range of auditory and non-auditory changes in laboratory rodents, at least [reviewed in: (25, 32)] which include the increased production of stress hormones [mice: (33, 34) rats: (35, 36)] cardiovascular damage [rats: (37)], histopathological changes in organs [rats: (38, 39)] decreased body weight [rats: (40)] and fertility [mice: (41, 42) rats: (39)], decrease in behavioral activity [mice: (43)] as well as expression of stress behaviors [e.g., canibalism in mice, (33)]. Such effects of acoustic stimuli can be life-long, with potential developmental, neural, genetic, and epigenetic consequences (20, 44). Such data from rodents have led to guidelines for laboratory animal care (15) that may or may not be relevant for laboratory birds (45).

There is now considerable evidence for the negative impacts of noise levels on wild birds (46–48) such as temporary physical damage to ears (49), stress responses including increased corticosterone metabolites (50, 51), telomere reduction (52), decreased in metabolic rate (53), decreased nestling size, and increased oxidative status (54), reduction in foraging (55), disturbance to vocal communication and risk perception (51, 56, 57) as well as decreased reproductive success [e.g., (51, 57, 58)]. As the hearing range for birds significantly differs from that of rodents (59), it seems plausible that husbandry practices for rodents are not necessarily applicable for the good management of birds, including songbirds (45). For example, the low-frequency fire alarms use in many animal facilities are adapted to emit sound at frequencies that alternate between 430 and 470 Hz, outside most rodents' auditory sensitive frequency range (60). However, these sounds are within the hearing range of several songbirds used in research, including zebra finches and canaries (61). For these birds, the “silent” fire alarm is not silent.

The impacts of such factors in a laboratory facility on bird behavior, however, have not been directly studied. To determine how an acute noise (low-frequency fire alarm) affected the behavior of laboratory zebra finches, we compared the differences in the duration of behaviors (such as general activity, stationary, foraging, preening and social behavior) from before and after birds were exposed to a routine low-frequency fire alarm test, as well as in comparison with a control (no fire alarm sound). It was hypothesized that the behavioral diversity and frequency performed by the birds would differ from before and after exposure.

## Materials and Methods

The work was conducted with approval from the Ethical Committee of the School of Biology at the University of St Andrews and from the Veterinary Ethical Review Committee of The Royal (Dick) School of Veterinary Studies at The University of Edinburgh (VERC Reference Number 29.18).

### Study Subjects

The subjects were 24 breeding pairs of zebra finches (*n* = 48), aged between 2 and 3 years that had been bred and kept in single-sex free-flight colony rooms in the St Mary's Animal Unit at the Bute Building, University of St Andrews.

### Experimental Procedure

In this facility a low-frequency fire alarm (Arrowmight Silentone^TM^, UK) simulation is part of the usual weekly laboratory routine. The test occurs every Monday at 13:00. According to the manufacturer the alarm activation generates a sound level of 97 dB (when measured at 450 mm) at a frequency between 430 and 470 Hz (62). During the weekly test, the fire alarm is rung for a period of 10–20 s, with the nearest alarm located 2.1 m away from the experimental room (Supplementary Figure 1). The experimental treatment of this experiment included this noise stimulus (see below for details of behavioral recording). The control treatment consisted of recording at the same time on a non-alarm day (Thursdays), where no noise was sounded.

**Figure 1:**
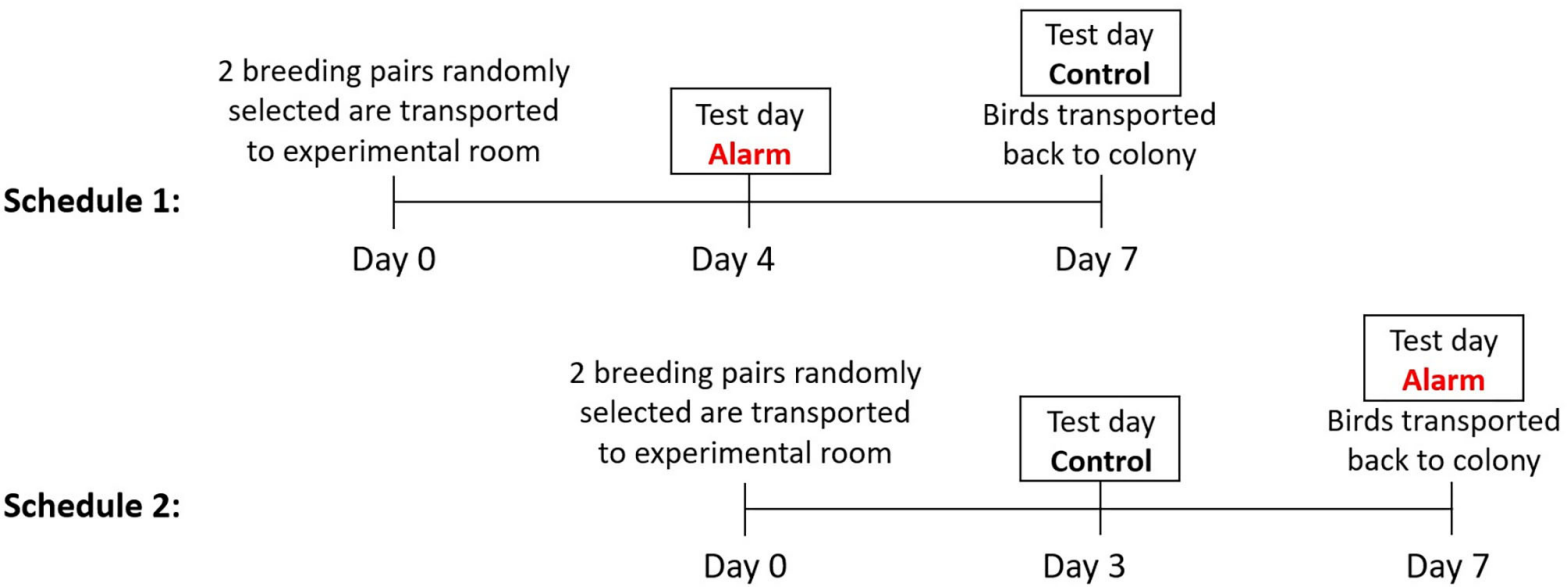
Timeline of experimental procedure. Birds subjected to Treatment 2 were transported to the experimental room for acclimatization on day 4 of the experiment for Treatment 1 birds. On day 7 all birds were returned to the colony rooms and new pairs were brought in. This staggered procedure was repeated until 24 pairs were filmed twice (for alarm and control).

**Figure 2:**
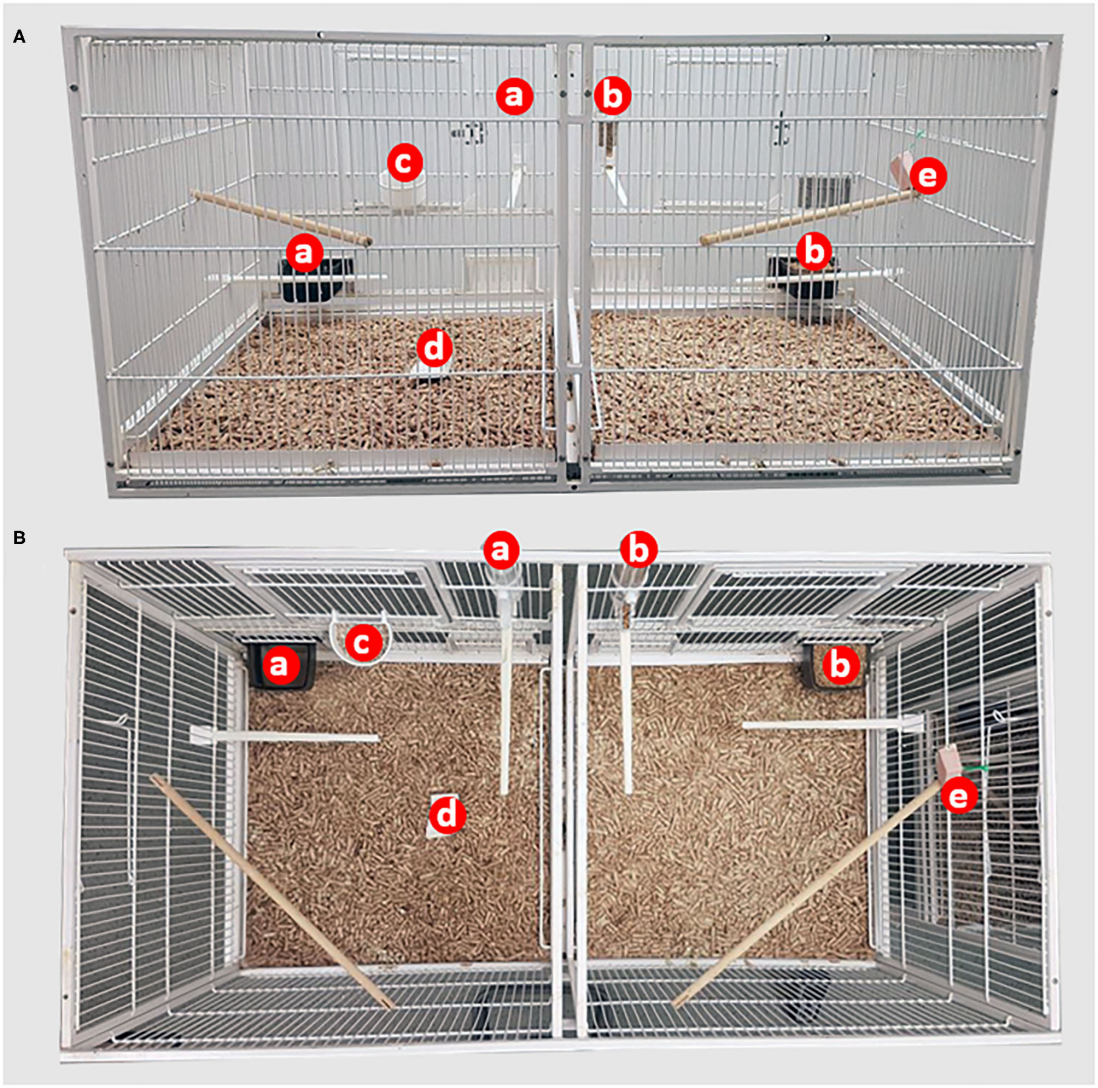
Back **(A)** and aerial **(B)** views of the layout of the experimental cage including the: **(a)** water bowl or hopper; **(b)** food bowl or hopper; **(c)** oyster shell grit bowl, **(d)** cuttlefish bone, and **(e)** mineral block.

Three to 4 days prior to testing eight birds were selected at random from the colony and were put into male and female pairs in order of capture. This period allowed acclimatization prior to the experimental period and preventing the birds being exposed to multiple alarm sounds. To balance for the order of exposure to either the control (no noise) or the fire alarm, the 24 pairs were equally distributed *via* random allocation to Treatment 1 or 2 (Figure 1). All birds were exposed to both treatments. Treatment 1 birds were exposed to the alarm noise on the 1st test day and the control on 2nd test day. Treatment 2 birds were exposed to the control on the 1st test day and the alarm noise on the 2nd test day. The pairing of closely-related birds (siblings and parents) was avoided, and a new individual was randomly selected from colony if such pairing occurred. Each pair was housed in a standardized experimental cage (100 × 50 × 50 cm), with six perches and with pressed wood pellets (Stovies Wood Pellets, Arbuthnott Wood Pellets Ltd., UK) as flooring (Figure 2). Birds could not see neighboring pairs (a white opaque sheet separated adjacent cages). All birds had *ad libitum* access to commercial bird seed mix (Johnston & Jeff Foreign Finch Seed, Johnston & Jeff Ltd., UK), supplemented water (Johnson's Vit-Min Drops for Cage Birds, Johnson's Veterinary Products Ltd., UK), cuttlefish, oyster shell grit (OYTA shells, Group Andersen, Spain), and a mineral block (Johnson's Iodised Condition Pek for Small Birds, Johnson's Veterinary Products Ltd., UK). They also received fresh spinach leaves twice a week (not on experimental days) as enrichment. The room was kept on a 14:10 h light:dark cycle, with temperatures between 18.8 and 20.7°C, and humidity levels that ranged between 37 and 55%.

Data collection consisted of video recording of the birds on test days. Filming took place between 12 and 2 p.m., corresponding to intervals of an hour before to an hour following the time of the scheduled fire alarm test. Four HD cameras (two Sony Handycams HDR-CX115, Sony Europe B.V., UK and two Cannon Legrias HFR46 / HFM52, Canon Inc., Japan) were used to record each one of the cages separately and were able to visualize all areas of each cage. When filming was complete for the four pairs, they were returned to the group-housing rooms. All husbandry (e.g., visual health inspection, cleaning, feeding, and water changes) occurred in the experimental room around 3–4 p.m., after filming was completed for the day. Birds were also moved at around this time of day.

Sound levels were recorded in the experimental room using a Thermosense HT-8852 Precision Sound Level Meter with Data Logger (Thermosense Limited, UK), which is sensitive to sound frequencies ranging from 31.5 Hz to 8 KHz. The Thermosense was placed on the floor in the middle of the experimental room, to provide the optimal overview of noise for all four cages, without compromising visualization of behavior. The equipment was adjusted with a fast time weighting (125 ms), for a more accurate estimation of peak sound levels; A-weighted (dBA), to take into account the decrease in hearing sensitivity of both birds and humans for frequencies lower than 1 kHz (63); and registered sound levels over the “automatic” level range (30–130 dB). The dBA levels were recorded every 0.5 s during the entire duration of the video recordings on all test days. Baseline recordings of dBA levels were performed in the empty experimental room prior to testing.

### Behavioral Scoring

Behavioral analysis was based on already-established ethograms for zebra finches (64, 65). Behaviors were broadly categorized into general activity, stationary, foraging, preening, social behavior, and brooding (Table 1). Ethological coding was performed using the event-logging software BORIS 6.3.6 (66). Durations of non-overlapping behavioral states were coded continuously starting an hour before and an hour after the stimulus (alarm/control) interval for both test days. This was done to generate activity budgets for the sample intervals based on the behaviors recorded (Table 1).

**Table 1:** Ethogram describing the behavioral categories behaviors used for the video behavioral coding.

**Behavior**	**Description**
Activity	General movement including: flying or moving (without the use of wings) from perch to perch, or perch to ground or moving sideways on a perch or hopping along the ground; OR water baths (diving, preening or flapping wings inside the water)
Stationary	The bird stays on the same spot for more than 5 s not performing any of the other defined behaviors
Foraging	Drinking water from any source OR eating either seeds, cuttlefish bone or grit OR scattering the floor in search of food or nest material If the bird pauses the behavior for a maximum of 3 s and then returned to it the whole duration of that activity was considered as the same “bout” of foraging.
Preening	Grooming the feathers and stretching the wings. If the bird pauses the behavior for a maximum of 3 s and then returned to it the whole duration of that activity was considered as the same “bout” of preening.
Social behavior	Allopreening (bird grooms or is groomed by partner) OR mating
Brooding	Bird sits/stands inside the nest quiet or tidying up nesting material or attempting to nest build.

*All behaviors were coded as state behaviors, with a start and finish time (obtaining duration data). As all behaviors were also mutually exclusive, the bird could perform only one of the behavioral categories at a time*.

### Statistical Analysis

Behavioral and sound pressure data were grouped into four 15-min intervals, using as reference the stimulus interval (alarm/control, time = 0): 30 BEFORE, 15 BEFORE, 15 AFTER, and 30 AFTER. The stimulus interval corresponded to the duration of the alarm test on Mondays and the corresponding time on the Thursday of the same week. Fifteen BEFORE and fifteen AFTER corresponded to the immediate 15 min before and after the stimulus interval, respectively. Thirty BEFORE and thirty AFTER corresponded to the further 15 min intervals within the selected time for analysis.

For the sound pressure data, the mean dBA of each 15 min interval was calculated for each separate day (alarm *n* = 8 and control *n* = 6). The mean dBA time series for each treatment, alarm and control, for when birds were present and absent from the experimental room.

For the behavioral data, the total duration of each behavior for each interval on the two different test days was recorded and differences between the 15 min intervals for each day (15 BEFORE minus 30 BEFORE, 15 AFTER minus 15 BEFORE, 30 AFTER minus 15 AFTER, and 30 AFTER minus 15 BEFORE) for the total duration of each behavior.

Multiple sequential sign tests were performed using Minitab 18® Statistical Software (67) and used to determine the intervals in which differences in behavior were observed after the alarm exposure. Those tests were corrected using a Holm-Bonferroni Sequential Correction (68) through Gaetano's EXCEL calculator (69). R (70) and lme4 (71) were then used to perform linear mixed-effects analyses of the relationship between the behavioral changes and treatment (alarm and control). Treatment, bird sex and the presence of a nest were entered as fixed effects and all interactions between them were included in the model (except for brooding behaviors, where nest was excluded from the model as only pairs that build performed that behavior). Pair ID and experimental schedule were included as nested random effects. This was done to control for multiple observations of birds housed in the same cage (pair) as well as the confounding impact schedule, as it could not be disassociated from other factors such as nest (unlike the birds in Schedule 1, the majority of birds in Schedule 2 built nests before the first experimental day, a difference confirmed with a Fisher's Exact-Test, *P* = 0.039, *N* = 23). A model was created for each behavioral category and model fitness was confirmed using the DHARMa package (72), the residuals of all models were in accordance to uniformity assumptions. Model *P*-values were calculated using Satterthwaite approximations of degrees of freedom provided by the lmerTest package (73).

To further explore the temporal effects on behavioral durations we divided each 15 min period into 5-min intervals (5A = 0–5 min; 10A = 5–10 min; and 15A = 10–15 min post-stimulus period). Within R (70) we used generalized linear mixed models [glmmTMB: (74)] to explore the relationship between the behavioral durations and interactions between the treatment and 5-min intervals. The family link function was set to negative binomial distribution (log transformation). In the minimum models, treatment and 5-min interval were entered as fixed effects, as well as the interaction between them. Pair ID and experimental schedule were included as nested random effects. Model fitness was confirmed using the DHARMa package (72), the residuals of all models were in accordance to uniformity assumptions.

Sound pressure data were analyzed using simple general linear models (GLM) to access the effect of treatment (alarm and control), interval (30 BEFORE, 15 BEFORE, 15 AFTER, and 30 AFTER) and the presence (or absence) of birds in the room on the dBA measures obtained. The stimulus interval was analyzed separately, evaluating only the impacts of treatment day and bird presence. The best-fitting models were selected on the basis of R^2^ values and lowest Second-order AIC (AICc), which was calculated using the package MuMIn (75). Model fitness was evaluated visually through diagnostic plots. Statistical differences between factors of the models were calculated using emmeans package (76) while pwr (77) was used to calculate the statistical power of the tests.

## Results

### Fire Alarm Duration and Sound Pressure

**Figure 3:**
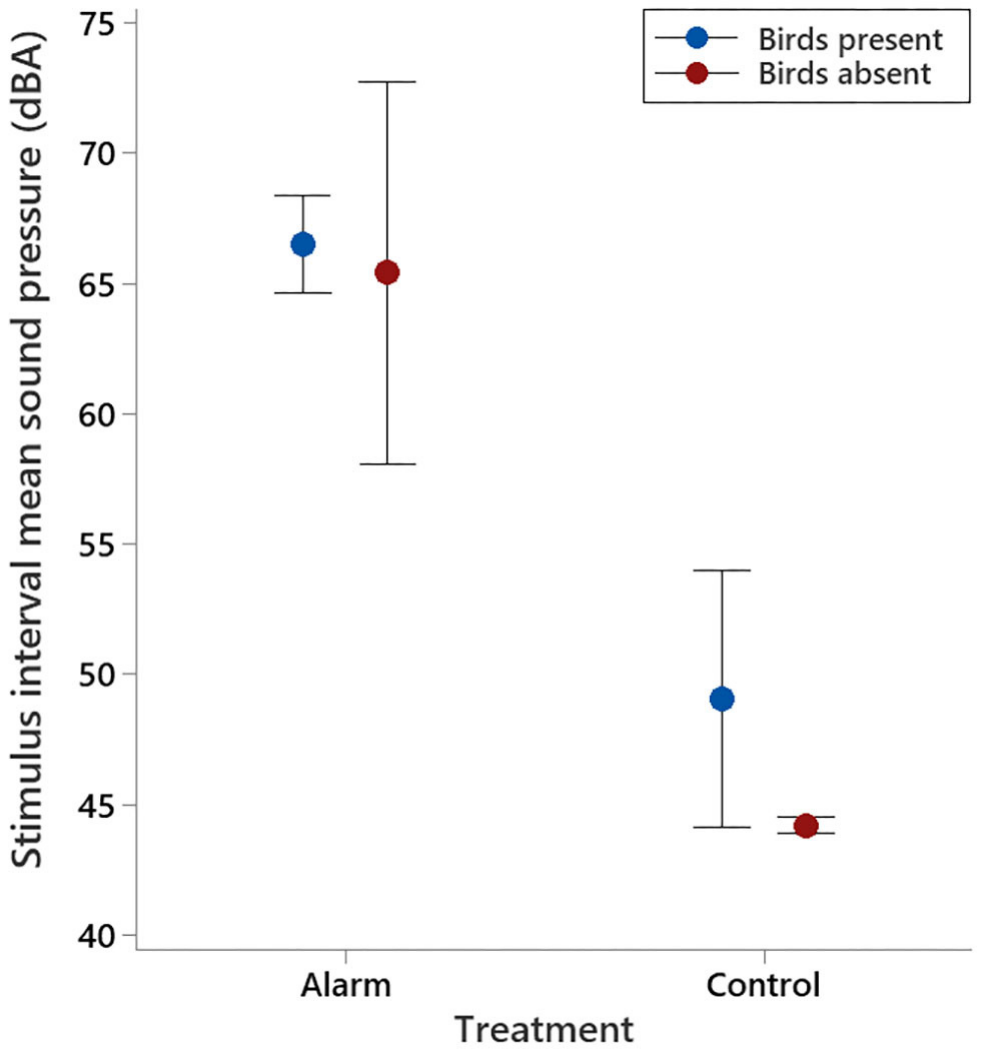
Mean (±95% CI) sound pressures observed in the experimental room for the 30 min prior to the stimulus interval and 30 min after, for both the alarm and control days, with birds present and absent.

Sound pressure levels in the experimental room during the stimulus interval were significantly higher on alarm test days than they were on control test days (F_1,15_ = 141.79, *P* < 0.001; Figure 3), irrespective of the presence of birds (F_1,15_ = 2.51, *P* = 0.13). During experimental procedures, the alarm sounded for an average of 17.7 ± 1.25 s.

### Sound Level Measurements of the Room During Subsequent Intervals

**Figure 4:**
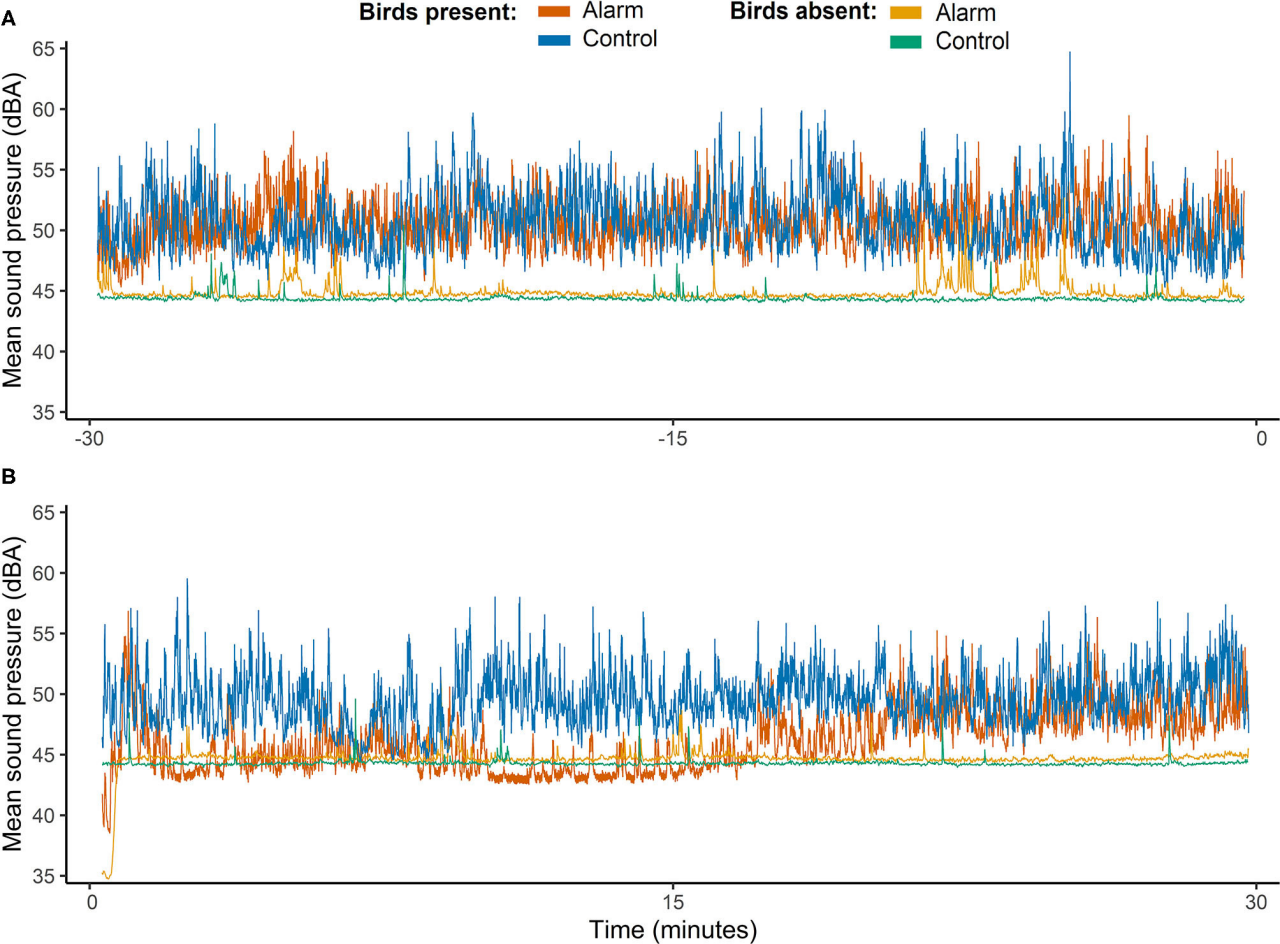
Time series of mean sound pressures (dBA) measured on each of the test days (alarm and control) and represented for days when birds were present and absent of the experimental room. Time axis shows a timescale in minutes of intervals before and after the stimulus interval (alarm and control). Negative numbers correspond to the minutes before the stimulus started **(A)** and positive values correspond to minutes after the stimulus interval finished **(B)**.

Comparisons of the room sound pressures during the intervals before and after the stimulus interval revealed that dBA pressures were higher and more variable when birds were present in the room (F_1,63_ = 29.11, *P* < 0.001; Figure 4). Raw sound pressure data indicated that when birds were present in the room dBA tended to drop right after the stimulus during the alarm treatment but not for the controls (see Figure 4). Although no interaction between test day and all intervals before and after was observed (F_3,63_ = 1.46, *P* = 0.23) when the full dataset was analyzed, the measurements during the 15 min after the stimulus tended to be lower on alarm test days than on control days (t_63_ = 1.88, *P* = 0.063; see Supplementary Figure 2).

**Figure 5:**
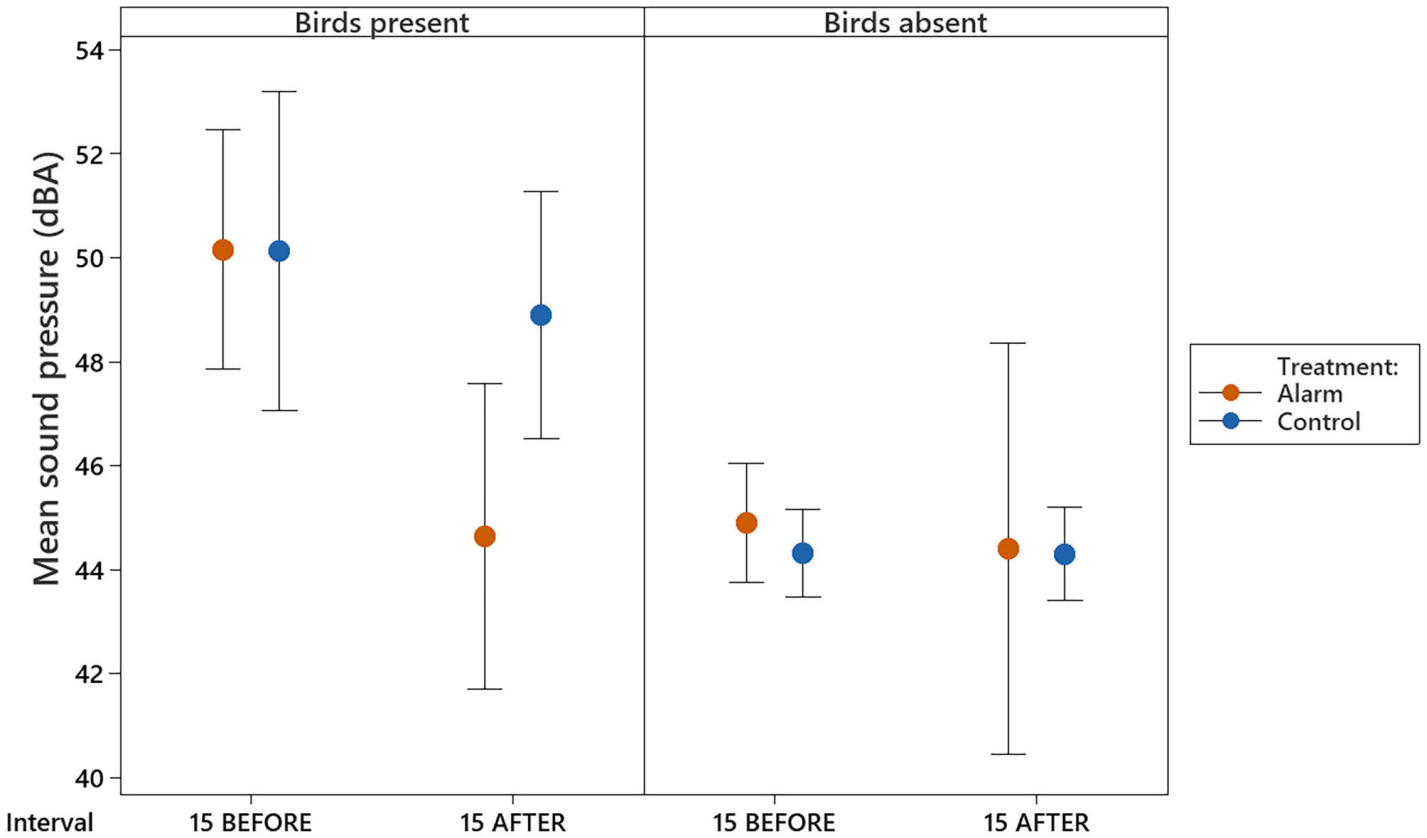
Mean (±95% CI) sound pressures observed in the experimental room for the intervals corresponding to the 15 min immediately before the stimulus (15 BEFORE) and 15 min immediately after (15 AFTER).

A direct comparison of the data of the intervals 15 BEFORE and 15 AFTER revealed that the levels remained lower 15 min immediately after the alarm than they were 15 min immediately before the alarm (t_31_ = −3.63, *P* = 0.019) and lower than control levels (t_31_ = −3.24, *P* = 0.049, Figure 5 and Supplementary Table 1). The control levels between the 15 min before and 15 min after the stimulus interval when birds were present did not differ (t_31_ = −0.67, *P* = 0.99). Moreover, the sound pressure levels on the 15 min after the stimulus on alarm days did not differ from the sound levels observed when the room was recorded without birds (Figure 5).

### Behavioral Observations

**Figure 6:**
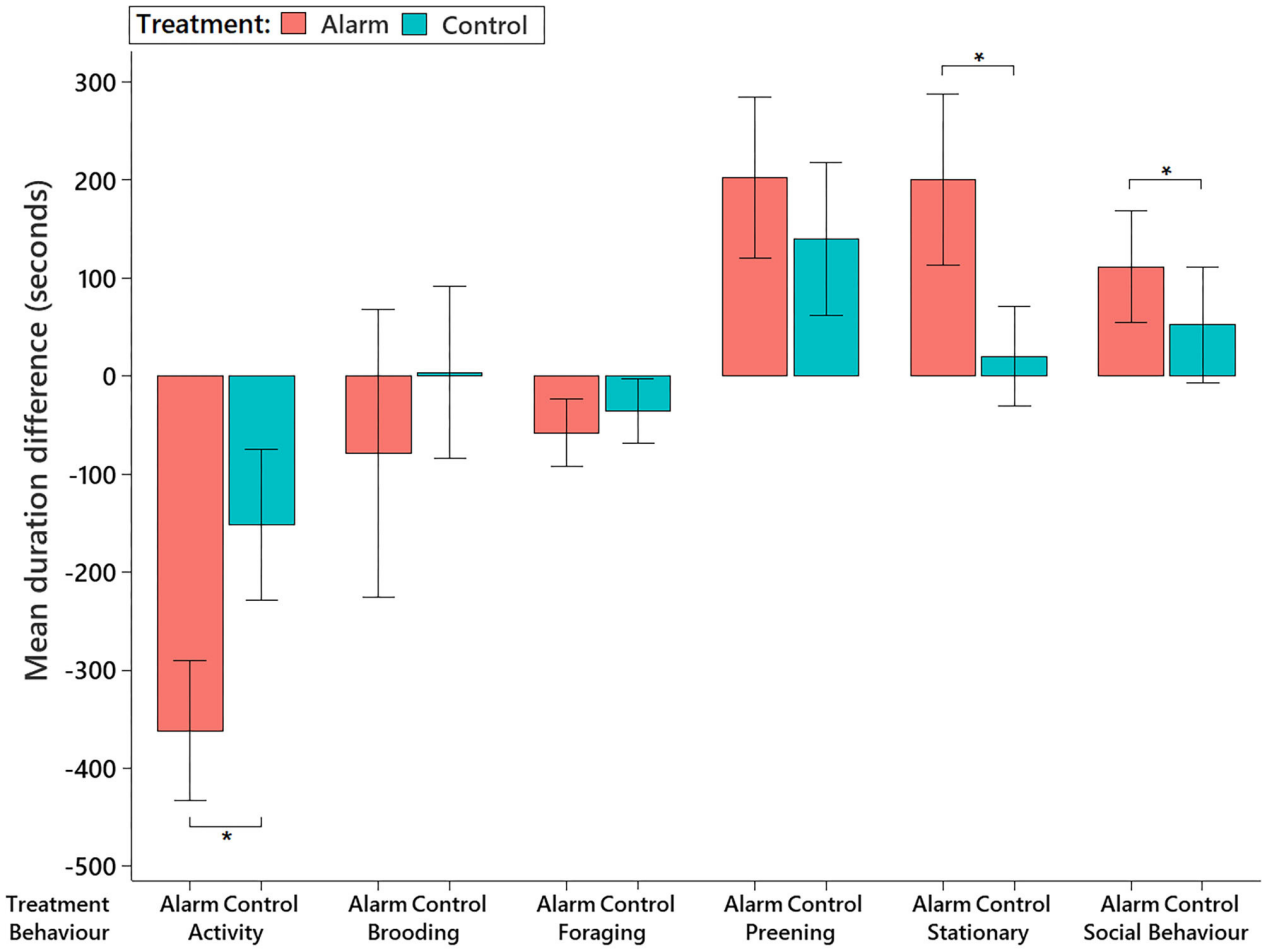
Mean (±95% CI) differences in time the birds spent (in seconds) performing different behaviors on the alarm and control days. Differences were calculated by subtracting the total duration of each behavior in the 15 min immediately before the stimulus interval from the duration observed on the 15 min subsequently after the stimulus. Behaviors marked with an asterisk *showed a significant difference (*P* < 0.05) on the behavioral pattern observed after the stimulus between the test days.

The behavioral impacts of the alarm were mainly observed during the intervals corresponding to the 15 min before and 15 min after the alarm (see Supplementary Table 2), as per observed in the sound level analysis. Thus, we focused on the behavioral differences observed between the 15 min immediately before the stimulus interval and the 15 min subsequently after the stimulus. During the 15 min immediately after the alarm, the birds significantly reduced their activity (F_1,59.96_ = 27.40, *P* < 0.001) and spent more time being stationary (F_1,61.11_ = 20.28, *P* < 0.001) than they were on control days (Figure 6 and also see Supplementary Figure 3). The birds also spent more time engaged in social interactions (mainly allopreening) after the alarm disturbance than they did in the control treatment (F_1,19.17_ = 32.70, *P* < 0.001). There were no differences in brooding (F_1,40_ = 1.44, *P* = 0.23), foraging (F_1,58.95_ = 1.25, *P* = 0.26), or preening (F_1,61.64_ = 1.08, *P* = 0.30) behaviors between the control and alarm (Figure 6).

**Figure 7:**
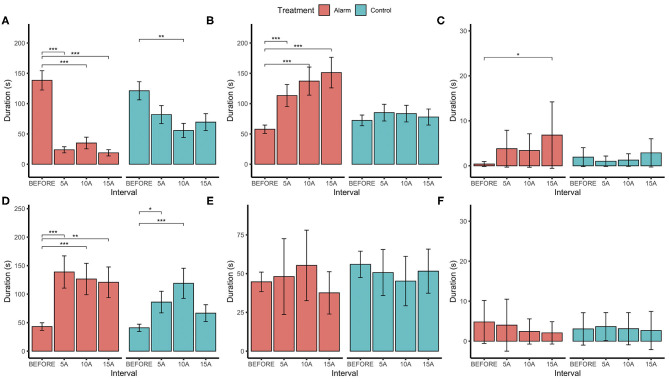
Estimated marginal means (±SE) for durations (in seconds) of **(A)** activity, **(B)** stationary, **(C)** social behaviors, **(D)** preening, **(E)** foraging, and **(F)** brooding behaviors before the stimulus (BEFORE) and in 5-min sample intervals after (5A = 0–5 min; 10A = 5–10 min; and 15A = 10–15 min) for both the alarm exposed and control groups. ^*^p < 0.05, ^**^p < 0.01, ^***^p < 0.001.

Assessment of 5-min subset samples within the 15-min intervals (through pairwise comparisons with the before interval) revealed that changes in the birds' behavior were maintained throughout the 15-min period after the alarm: general activity ($\chi_{3,545}^{2}$ = 34.82, *P* < 0.001), stationary ($\chi_{3,545}^{2}$ = 21.17, *P* < 0.001), and preening behaviors ($\chi_{3,545}^{2}$ = 3.53, *P* = 0.317; Figure 7). Although the alarm treatment birds performed social behaviors for longer than did the control group ($\chi_{3,545}^{2}$ = 8.38, *P* < 0.038), there was substantial variation between pairs. This resulted in a difference only between 15 BEFORE and the 15 AFTER being revealed in the pairwise comparisons. Sampling 5 min interval periods revealed no differences in foraging and brooding behavior.

## Discussion

Exposure to a low-frequency fire alarm induced behavioral changes in laboratory zebra finches: birds decreased all movements and spent more time on social behaviors (allopreening). Sound pressure level analysis also revealed that the birds themselves generated less noise after alarm exposure. These data provide the first evidence for a welfare impact of low-frequency fire alarms in animal laboratories on zebra finches.

Low-frequency alarms have been designed primarily to minimize the stress responses and reduce physiological impacts in laboratory rodents (60) and the majority of guidelines regarding optimizing welfare are addressed to two of the most common used animals in research, mice and rats (78, 79). The sound pressure levels for the low-frequency alarm are within the hearing range of zebra finches (61, 63), confirming the birds are able to hear the alarm sound caused by the equipment. When the alarm sounded, birds immediately flew to a perch and either sat motionless or start to preen. That this change in behavior was caused by the alarm is confirmed by the absence of a disruption to the birds' behavior during control days. This reduction in movement could be evidence of a fear response as a decrease in locomotor activity has also been documented in >20 mammal prey species (80, 81) and other passerine birds [e.g., pied flycatchers, *Ficedula hypoleuca*: (82)].

Mice also respond to exposure to a low-frequency fire alarm for 60 s by reducing activity, a response attributed to startle behaviors (43), although the alarm is considered to be inaudible to them (60). Startle responses are defined as fast responses to sudden, intense stimuli that may serve as a protective reaction against injury from a predator and as preparation for a fight/flight response (83, 84). Such reactions are associated with aversive stimuli and may induce a state of fear or anxiety (84), they are also known to be potentialized by fear-conditioning, when a cue predicts an aversive stimuli (84, 85). As latency in movement in face of a threat has also been associated with startle responses in birds [blue jays, *Cyanocitta eristata:* (86)], it seems plausible that the zebra finches' responses to the fire alarm can be considered as startle behaviors. Because the zebra finches remained still and quiet for a much longer period than the mice reported in Povroznik et al. (43), it may be that the birds are even more affected by the alarm disturbance than are mice, perhaps because zebra finches have a higher auditory sensitivity in lower frequencies than mice (59, 60, 63).

The increase in social behaviors after alarm exposure might also be associated with fear responses. Huddling in face of a fearful situation has been reported in several different species [e.g., ungulates: (87); humans: (88); rats: (81)], including birds [penguins: (89)]. The choice of the zebra finches to perch in the middle of the cage in response to the alarm is similar to the reactions of zebra finches to an open-field test (90). Rifá et al. (90) argued that the birds preferred this position as it allowed for the broadest possible field of vision.

The decreased sound pressure observed in the experimental room after alarm exposure is yet more evidence for the behavioral effect of the alarm over the birds' behavior. Zebra finches are known for their highly variable vocal repertoire and constant vocal contact (91–93). It is probable that the fluctuating pattern of sound measurements recorded in the presence of birds was caused by the birds themselves, from actual vocalizations in combination with activity in the cage, rather than other environmental sources. This interpretation is supported by the lower and less variable levels of sound in the empty room. The decreased sound pressure levels after the alarm to levels similar to those observed in the absence of the animals, suggests that the animals decreased activity and communication after being exposed to the alarm disturbance. The absence of such an effect during control test days, when the dBA levels continued to fluctuate as before the stimulus interval, is evidence that the observed effects were indeed due to the alarm disturbance. Sudden drops in vocalizations can be interpreted as a signal of danger (94, 95) or as an attempt at concealment in face of a predation danger (80, 96).

A behavioral characterization of predator model response for zebra finches was recently attempted by Butler et al. (93). The experiment used an auditory (wing flap sounds) and two virtually animated visual (flyby and loom) model collared sparrowhawk stimuli to analyse behavioral responses, alarm call presence and call rate changes in captive zebra finches. The zebra finches performed anti-predator flight behaviors toward the looming hawk stimulus and startle/attention behaviors in response to the less threatening wing flap sounds and flyby stimuli. However, the birds did not present any characteristic alarm calls or change their call rate and structure at either 30 s or at 30 min after exposure to any of the three stimuli presented. That result contrasts with the marked decrease in vocal communication observed in the present study, after the fire alarm. Butler et al. (93) identified that the zebra finches' response was stronger toward the most threatening stimulus (looming hawk). Perhaps the difference in response observed in the two experiments could be attributed to the level of perceived threat of the stimuli presented. The fire alarm might have been perceived as higher risk and more stressful than the predator cues presented by Butler et al. (93).

It is, perhaps, surprising that the birds responded as they did, given all the birds were hatched, and had lived, in the facility for ~3 years. One might have expected them to have been habituated to the weekly alarm. Although plausible, the behavioral responses that we observed suggest this is not the case and raises concerns about the suitability of such equipment for the use in animal facilities where birds are housed. Although the low-frequency alarm is considered as safe for use near laboratory rodents [(60); but see (43)], it appears to be a disturbing stimulus for zebra finches.

Repeated aversive noise exposure could produce morphological and biochemical effects in noradrenaline and adrenaline cells (97) and continuous exposure to predatory threats has been shown to induce chronic stress and anxiety (98–101). These long-lasting stress responses can even induce cross-generational consequences for animal welfare (44). Further concerns thus could be raised over the possible cumulative effect of the repeated exposure of the birds to the fire alarm disturbance, which has not been investigated to date.

The changes in behavior induced by the alarm caused variation in the birds' behavioral patterns across the week, which in turn, could potentially impact the results of behavioral experiments (102). Physiological consequences of the noise stress can also produce similar effects, confounding research outcomes and possible reproducibility (7, 9, 25, 102, 103). Such considerations are important for the management of passerine birds in research facilities, as decisions on the “best” housing conditions of such animals are mostly based on expert and experienced keepers' advice (104).

These data provide a basis for the assessment of the acute welfare impacts of ambient noise in laboratory zebra finches. They also suggest that there may be physiological and longer-term behavioral impacts on bird welfare of the use of low-frequency alarms. As the low-frequency alarm noise had an evident effect on the birds' behavior, we suggest the use of low-frequency sound alarms in laboratory bird facilities may have both detrimental effects on animal welfare (acute behavioral distress response) and on the reliability and reproducibility of research outputs.

## Data Availability Statement

The raw data supporting the conclusions of this article will be made available by the authors, without undue reservation, to any qualified researcher upon request.

## Ethics Statement

The animal study was reviewed and approved by the Ethical Committee of the School of Biology at the University of St Andrews and the Veterinary Ethical Review Committee of The Royal (Dick) School of Veterinary Studies at The University of Edinburgh (VERC Reference Number 29.18).

## Author Contributions

TC and SH contributed to the conception and design of the study. TC did the laboratory practical work, data collection, and wrote the first draft of the manuscript. TC organized the database and performed the statistical analysis with guidance from both JM and SH. JM and SH made substantial edits to the manuscript, both in substance and style. All authors contributed to manuscript revision, read, and approved the submitted version.

## Conflict of Interest

The authors declare that the research was conducted in the absence of any commercial or financial relationships that could be construed as a potential conflict of interest.
