# Supplementary material for: The Impact of Acute Loud Noise on the Behavior of Laboratory Birds
Source: Front Vet Sci. 2021 Jan 6;7:607632. doi: 10.3389/fvets.2020.607632 (PMC7815526; doi:10.3389/fvets.2020.607632)
Supplement: Supplementary file 1 [file Data_Sheet_1.docx]

Supplementary Material

# Supplementary Data

**
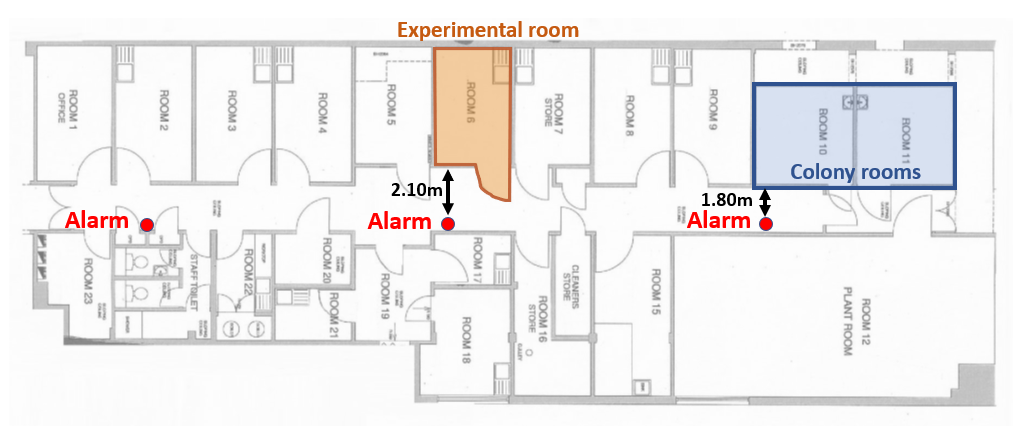
**

**Supplementary Figure 1.** Floor plan of the bird facility at St Mary’s Animal Unit (SMAU). The studied pairs were housed in Room 6 (marked in orange). The locations of the fire alarm devices along the corridor are shown as red dots (opposite Rooms 2, 6, and 10). The free flight colony rooms where birds were usually housed is located at a comparable proximity to the fire alarm as the experimental room. Adapted with permission from SMAU.


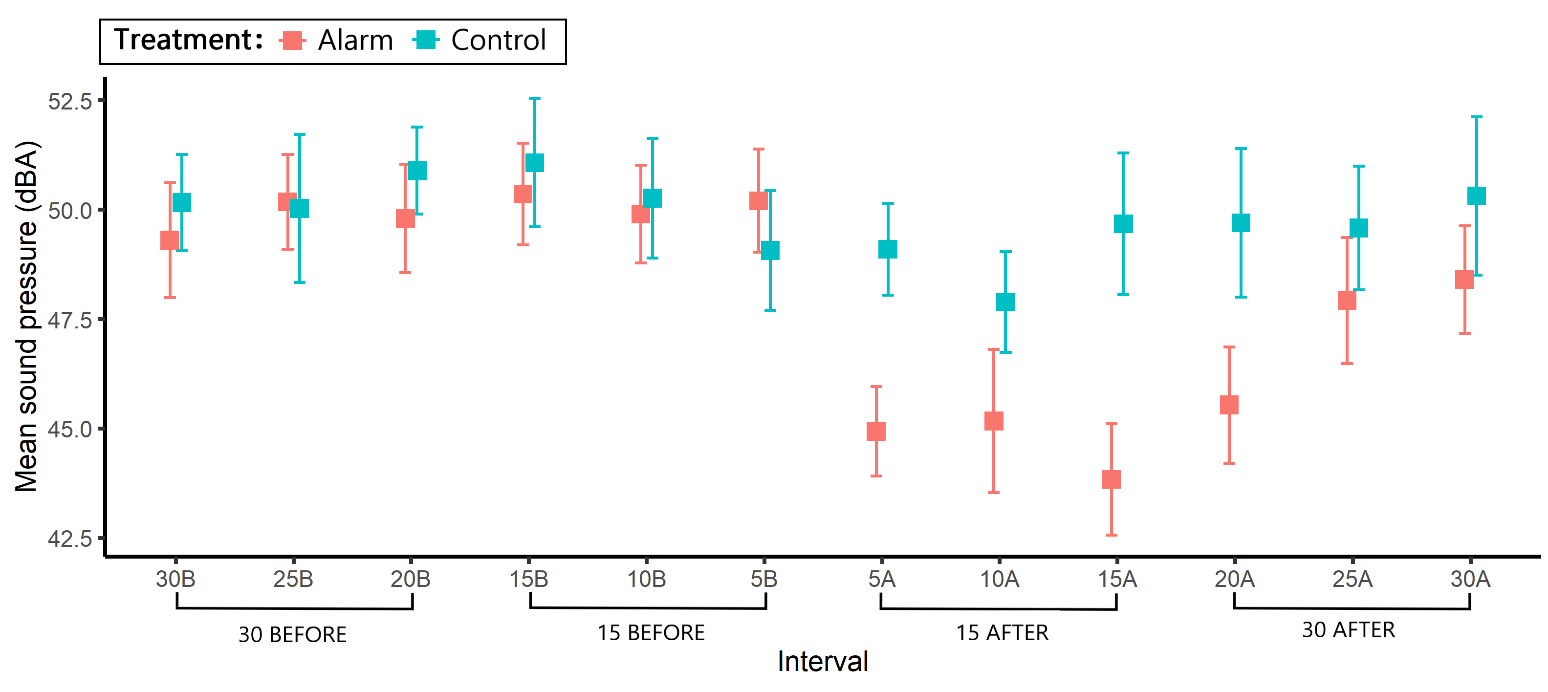


**Supplementary Figure 2.** Sound pressure means (± SE) calculated for different intervals for each test day (alarm/control) when birds were present in the experimental room.

**Supplementary Table 1.** Summary of Post hoc comparisons using Turkey's test for dBA data. Samples correspond to the intervals 15 BEFORE and 15 AFTER the stimulus interval on control and alarm test days. Experimental (days with birds present – Birds in “Yes”) and empty room analysis (Birds in “No”) are included.

| Treatment | Interval | Birds  in | Treatment | Interval | Birds  in | Estimate | SE | Df | T Ratio | P Value |
| --- | --- | --- | --- | --- | --- | --- | --- | --- | --- | --- |
| Control | 15 AFTER | No | Alarm | 15 AFTER | No | 3.3433317 | 1.316336 | 31 | 2.540 | 0.2167 |
| Control | 15 AFTER | No | Control | 15 BEFORE | No | -0.9314825 | 1.386298 | 31 | -0.672 | 0.9972 |
| Control | 15 AFTER | No | Alarm | 15 BEFORE | No | -1.1629735 | 1.316336 | 31 | -0.883 | 0.9855 |
| Control | 15 AFTER | No | Control | 15 AFTER | Yes | -3.9366684 | 1.113501 | 31 | -3.535 | **0.0251*** |
| Control | 15 AFTER | No | Alarm | 15 AFTER | Yes | -0.5933367 | 1.687790 | 31 | -0.352 | >0.999 |
| Control | 15 AFTER | No | Control | 15 BEFORE | Yes | -4.8681509 | 1.778119 | 31 | -2.738 | 0.1491 |
| Control | 15 AFTER | No | Alarm | 15 BEFORE | Yes | -5.0996419 | 1.687790 | 31 | -3.021 | 0.0827 |
| Alarm | 15 AFTER | No | Control | 15 BEFORE | No | -4.2748142 | 1.316336 | 31 | -3.248 | **0.0499*** |
| Alarm | 15 AFTER | No | Alarm | 15 BEFORE | No | -4.5063052 | 1.239943 | 31 | -3.634 | **0.0197*** |
| Alarm | 15 AFTER | No | Control | 15 AFTER | Yes | -7.2800001 | 1.759720 | 31 | -4.137 | **0.0054*** |
| Alarm | 15 AFTER | No | Alarm | 15 AFTER | Yes | -3.9366684 | 1.113501 | 31 | -3.535 | **0.0251*** |
| Alarm | 15 AFTER | No | Control | 15 BEFORE | Yes | -8.2114826 | 1.759720 | 31 | -4.666 | **0.0013*** |
| Alarm | 15 AFTER | No | Alarm | 15 BEFORE | Yes | -8.4429736 | 1.666536 | 31 | -5.066 | **0.0004*** |
| Control | 15 BEFORE | No | Alarm | 15 BEFORE | No | -0.2314911 | 1.316336 | 31 | -0.176 | >0.999 |
| Control | 15 BEFORE | No | Control | 15 AFTER | Yes | -3.0051859 | 1.778119 | 31 | -1.690 | 0.6932 |
| Control | 15 BEFORE | No | Alarm | 15 AFTER | Yes | 0.3381458 | 1.687790 | 31 | 0.200 | >0.999 |
| Control | 15 BEFORE | No | Control | 15 BEFORE | Yes | -3.9366684 | 1.113501 | 31 | -3.535 | **0.0251*** |
| Control | 15 BEFORE | No | Alarm | 15 BEFORE | Yes | -4.1681595 | 1.687790 | 31 | -2.470 | 0.2455 |
| Alarm | 15 BEFORE | No | Control | 15 AFTER | Yes | -2.7736949 | 1.759720 | 31 | -1.576 | 0.7602 |
| Alarm | 15 BEFORE | No | Alarm | 15 AFTER | Yes | 0.5696368 | 1.666536 | 31 | 0.342 | >0.999 |
| Alarm | 15 BEFORE | No | Control | 15 BEFORE | Yes | -3.7051773 | 1.759720 | 31 | -2.106 | 0.4336 |
| Alarm | 15 BEFORE | No | Alarm | 15 BEFORE | Yes | -3.9366684 | 1.113501 | 31 | -3.535 | **0.0251*** |
| Control | 15 AFTER | Yes | Alarm | 15 AFTER | Yes | 3.3433317 | 1.316336 | 31 | 2.540 | 0.2167 |
| Control | 15 AFTER | Yes | Control | 15 BEFORE | Yes | -0.9314825 | 1.386298 | 31 | -0.672 | 0.9972 |
| Control | 15 AFTER | Yes | Alarm | 15 BEFORE | Yes | -1.1629735 | 1.316336 | 31 | -0.883 | 0.9855 |
| Alarm | 15 AFTER | Yes | Control | 15 BEFORE | Yes | -4.2748142 | 1.316336 | 31 | -3.248 | **0.0499*** |
| Alarm | 15 AFTER | Yes | Alarm | 15 BEFORE | Yes | -4.5063052 | 1.239943 | 31 | -3.634 | **0.0197*** |
| Control | 15 BEFORE | Yes | Alarm | 15 BEFORE | Yes | -0.2314911 | 1.316336 | 31 | -0.176 | >0.999 |
| * indicates statistically significant (P<0.05) differences between samples. | | | | | | | | | | |

**Supplementary Table 2.** Results of the sequential sign tests performed to select significant intervals when changes in behaviour patterns could be seen during alarm days. Samples correspond to comparisons of behaviour durations between before(B) and after(A) intervals (later interval minus previous interval) for alarm days.

| Sample | Number of Values | | | P' |
| --- | --- | --- | --- | --- |
|  | < 0 | = 0 | > 0 |  |
| Activity 15B-**15A** | 46 | 0 | 0 | **< 0.001*** |
| Activity **15A**-30A | 10 | 0 | 34 | **< 0.001*** |
| Activity 15B-30A | 37 | 0 | 9 | **< 0.001*** |
| Preening 15B-**15A** | 10 | 0 | 36 | **< 0.001*** |
| Foraging 15B-**15A** | 37 | 0 | 7 | **< 0.001*** |
| Foraging **15A**-30A | 9 | 0 | 32 | **< 0.001*** |
| Preening **15A**-30A | 35 | 0 | 11 | **0.018*** |
| Brooding 15B-**15A** | 17 | 0 | 4 | 0.119 |
| Stationary **15A**-30A | 32 | 0 | 14 | 0.176 |
| Stationary 15B-**15A** | 15 | 0 | 31 | 0.390 |
| Brooding **15A**-30A | 5 | 0 | 16 | 0.390 |
| Social Behaviour **15A**-30A | 14 | 0 | 4 | 0.403 |
| Social Behaviour 15B-**15A** | 6 | 0 | 16 | 0.624 |
| Social Behaviour 15B-30A | 6 | 0 | 12 | >0.999 |
| Stationary 30B-15B | 19 | 0 | 27 | >0.999 |
| Stationary 15B-30A | 19 | 0 | 27 | >0.999 |
| Social Behaviour 30B-15B | 10 | 0 | 6 | >0.999 |
| Foraging 30B-15B | 20 | 0 | 26 | >0.999 |
| Brooding 15B-30A | 9 | 0 | 13 | >0.999 |
| Preening 30B-15B | 21 | 0 | 25 | >0.999 |
| Foraging 15B-30A | 20 | 0 | 23 | >0.999 |
| Preening 15B-30A | 24 | 0 | 22 | >0.999 |
| Activity 30B-15B | 23 | 0 | 23 | >0.999 |
| Brooding 30B-15B | 10 | 0 | 11 | >0.999 |
| ** indicates statistically significant (P<0.05) samples.* | | | | |


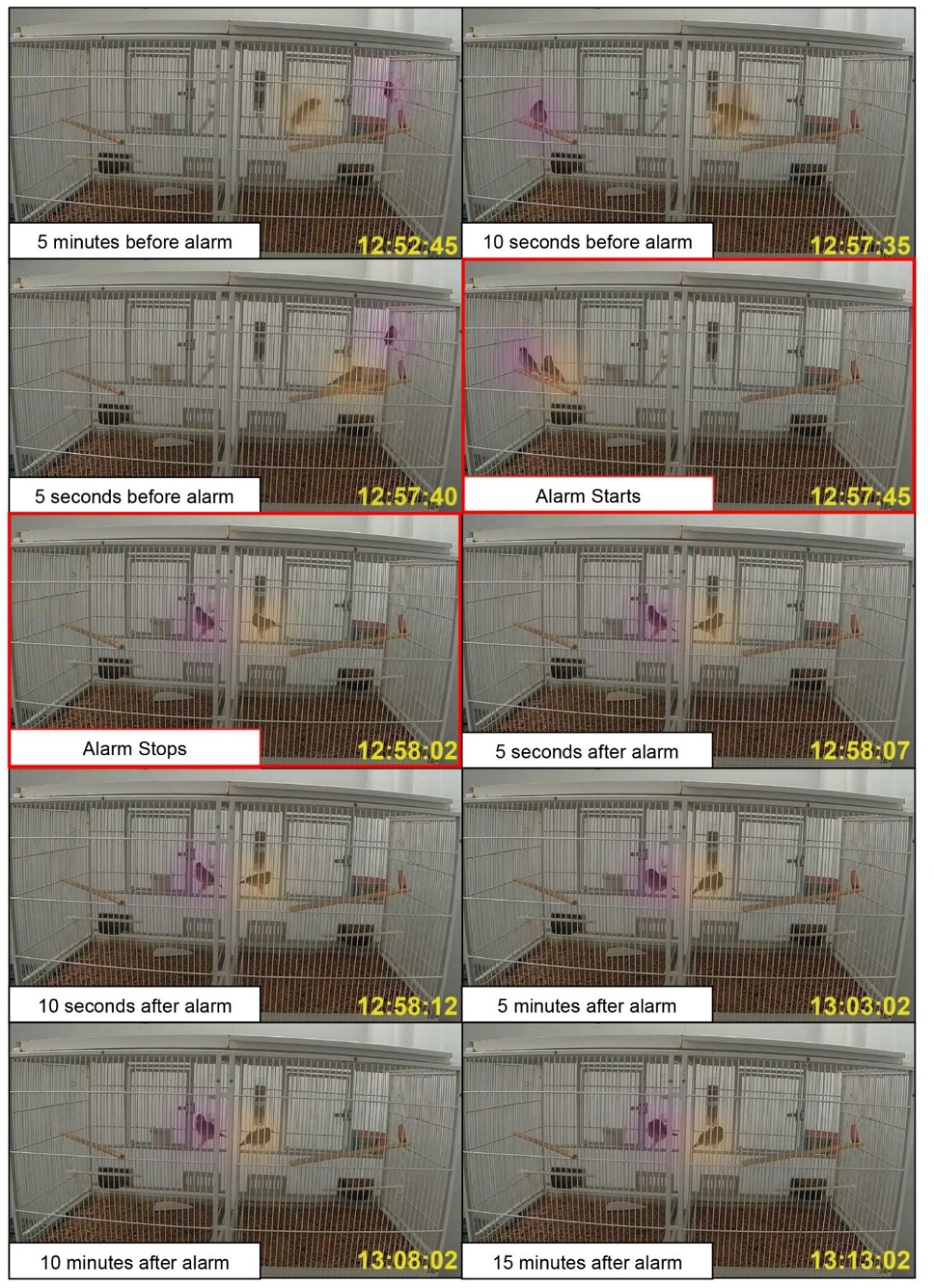


**Supplementary Figure 3.** Example of the behavioural pattern observed for experimental birds on Mondays (alarm day). Birds (male highlighted in yellow and female highlighted in purple) were highly active on the intervals before the alarm was sounded. When the alarm was sounded birds immediately stopped any activity and flew to a perch in the middle of the cage. They then sat motionless and preened intermittently, not moving from the spot they moved to after the alarm. The alarm interval was highlighted in red.
